# Thymol-Loaded Eudragit RS30D Cationic Nanoparticles-Based Hydrogels for Topical Application in Wounds: In Vitro and In Vivo Evaluation

**DOI:** 10.3390/pharmaceutics15010019

**Published:** 2022-12-21

**Authors:** Amira Mohamed Mohsen, Yosra Ibrahim Nagy, Amr M. Shehabeldine, Mona M. Okba

**Affiliations:** 1 Pharmaceutical Technology Department, National Research Centre, El-Buhouth Street, Dokki, Cairo 12622, Egypt; 2 Department of Microbiology and Immunology, Faculty of Pharmacy, Cairo University, Cairo 11562, Egypt; 3 Department of Botany and Microbiology, Faculty of Science (Boys), Al-Azhar University, Nasr City, Cairo 11884, Egypt; 4 Department of Pharmacognosy, Faculty of Pharmacy, Cairo University, Kasr El-Ainy, Cairo 11562, Egypt

**Keywords:** thymol, Eudragit RS30D, cationic polymeric nanoparticles, hydrogel, skin retention, wound healing

## Abstract

Natural medicines formulated using nanotechnology-based systems are a rich source of new wound-treating therapeutics. This study aims to develop thymol-loaded cationic polymeric nanoparticles (CPNPs) to enhance the skin retention and wound healing efficacy of thymol. The developed materials exhibited entrapment efficiencies of 56.58 to 68.97%, particle sizes of 36.30 to 99.41 nm, and positively charged zeta potential. In Vitro sustained release of thymol up to 24 h was achieved. Selected thymol CPNPs (F5 and C2) were mixed with methylcellulose to form hydrogels (GF5 and GC2). An In Vivo skin-retention study revealed that GF5 and GC2 showed 3.3- and 3.6-fold higher retention than free thymol, respectively. An In Vitro scratch-wound healing assay revealed a significant acceleration in wound closure at 24 h by 58.09% (GF5) and 57.45% (GC2). The potential for free thymol hydrogel, GF5, and GC2 to combat MRSA in a murine skin model was evaluated. The bacterial counts, recovered from skin lesions and the spleen, were assessed. Although a significant reduction in the bacterial counts recovered from the skin lesions was shown by all three formulations, only GF5 and GC2 were able to reduce the bacterial dissemination to the spleen. Thus, our study suggests that Eudragit RS30D nanoparticles-based hydrogels are a potential delivery system for enhancing thymol skin retention and wound healing activity.

## 1. Introduction

Wound healing has experienced extensive research in order to ensure function preservation while achieving rapid healing and minimal scarring [1]. Colonization with an infectious agent is the most common problem encountered with wound closure. Although the bacteria that make up the skin microbiota are helpful in avoiding the colonization of other diseases, they can impede the healing process once they reach a certain threshold at the site of a lesion [2]. The most frequently discovered colonizing pathogens that affect the earliest stages of wound healing are *Staphylococcus aureus* and methicillin-resistant *Staphylococcus aureus* (MRSA) [3]. The majority of current wound-healing therapies don’t produce satisfactory therapeutic results, either physically or functionally. Hence, it is essential to use a suitable antimicrobial-associated wound treatment to stop bacterial infections and promote the natural healing process.

Many pure compounds of natural origin are well-documented in managing several diseases [4]. Thymol (5-methyl-2-isopropyphenol) is the primary active constituent in thyme and oregano essential oils. It has been shown to have several bioactivities, including antioxidant, antispasmodic, wound-healing, and anti-inflammatory activities [5,6]. Moreover, the antibacterial activity of thymol is also superior to those of other essential oil constituents such as carvacrol, cinnamic acid, eugenol, and diacetyl [7]. Thymol is also known to possess antimicrobial activity against a wide array of pathogenic microorganisms including *Staphylococcus aureus* [8,9,10]. Regardless of geographical area, patient age, or climate, *S. aureus* is, worldwide, the most common pathogen involved in skin infections [11]. Abscesses incidence is increasing and community-acquired MRSA, mainly causing skin infections, has become common [11,12]. However, thymol’s limited water solubility, high volatility, and potent aromatic odor might result in a significant hindrance to its use [13]. This motivated the authors to evaluate the effectiveness of nano-formulated thymol preparations.

The introduction of nanotechnology has allowed scientists to extensively investigate more effective treatments [14]. Different nanosystems have been developed to incorporate a variety of medications in order to improve their activity and bioavailability [15,16,17]. Nanoparticles (NPs) can be defined as structures displaying sizes that range from 1 to 100 nm [18]. The goal of using nanomaterial-based methods is to develop novel antibacterial alternatives that might destroy different harmful microorganisms. Because of its diverse physicochemical characteristics, nanotechnology is a dependable study area for treatments for wound healing. It is simple to modify the biochemical properties of nanoparticles such as hydrophobicity and capability of tissue penetration to deeper layers, by altering the material type and particle size, as well as the nanoparticle electrical charge [19]. Due to their stability and surface modification selectivity, polymeric nanoparticles have gained a lot of interest [20]. Several polymers have been studied to develop cationic polymeric nanoparticles (CPNPs). Eudragit RL30D and Eudragit RS30D are widely employed, along with other acrylic polymers, as sustained-release polymers. They are cationic copolymers comprising quaternary ammonium compounds [21,22]. Eudragit RS30D exhibits a more hydrophobic nature and is broadly employed to control the drug release rate. The physicochemical features of polymers are an important aspect when choosing them for a specific release pattern [22,23].

Antimicrobial drugs based on cationic chemicals are likely to be successful [24]. Of all the surfactants, quaternary ammonium compounds (QACs) are the appropriate choice as they are known to be the most useful antiseptics and disinfectants [25]. Antimicrobial cationic surfactants with a hydrophobic alkyl chain and a hydrophilic quaternary ammonium group have high bactericidal activity and are widely used for sanitation and disinfection in a number of settings, including food processing and hospitals [26]. Antimicrobial cationic surfactants, such as cetylpyridinium chloride, benzalkonium chloride (BAC), and cetyltrimethylammonium bromide (CTAB), are particularly safe, and of cost-effective high productivity [27]. CTAB, a positively charged surfactant, is commonly used in nanoparticle formulations [28]. Therefore, in this study, it was selected to investigate its effect on the efficacy of the developed thymol-loaded Eudragit NPs.

This study was designed to develop thymol-loaded CPNPs, which were further characterized. Several CPNP formulations were fabricated employing two types of polymers (Eudragit RL30D and Eudragit RS30D), and different drug:polymer (D:P) weight ratios. The optimized thymol-loaded CPNP formulations were further loaded in methyl cellulose hydrogels for topical administration. The developed hydrogels were characterized via several characterization methods and were further examined for enhancing the therapeutic efficacy of thymol. The goal of the current study was to fabricate thymol CPNP hydrogels to enhance the skin retention and wound healing efficacy of thymol against MRSA skin infection. The newly developed formulations are expected to control thymol release, improve its activity, and enhance patient compliance.

## 2. Materials and Methods

### 2.1. Materials

#### 2.1.1. Chemicals

Thymol (L33300) was procured from the El-Gomhouria pharmaceutical company, Zeitoun, Cairo.

Eudragit RL-30D^®^ and Eudragit RS-30D^®^ were kindly donated by Evonik Rohm GmbH, Pharma Polymers, Germany. Kolliphor^®^ P188 (P-188), acetone, porcine mucin, and Mesocell (methyl cellulose) were purchased from Sigma-Aldrich Chemical Co., St. Louis, MO, USA. Methanol was purchased from Fisher Scientific, Leicestershire, UK. Cetyl trimethyl ammonium bromide (CTAB) was procured from Loba Chemie, Mumbai, India. Other chemicals were of analytical grade.

#### 2.1.2. Animals

Female BALB/c mice, six-to-eight weeks old, weighing 20–22 g were used in In Vivo studies. Mice were kept in polyethylene cages and fed a standardized laboratory diet with free access to water. Mice were housed in 12 h dark/light cycles. Animal procedures were carried out according to the institutional ethical and regulatory guidelines and approved by the Research Ethics Committee of the Faculty of Pharmacy, Cairo University, Egypt [approval No. (MI 3138)].

### 2.2. Preparation of Thymol-Loaded CPNPs

Thymol-loaded CPNPs were fabricated by the nanoprecipitation method, as described by Fessi et. al. [29]. Two types of Eudragit materials, namely, Eudragit RS-30D and Eudragit RL-30D were employed as cationic polymers. P-188 was fixed at 0.5 (*w*/*v*%) in all formulations. The cationic stabilizer CTAB was incorporated in formulations C1 and C2 at a concentration of 0.25 (*w/v*%) to investigate its antimicrobial effect. Briefly, accurately weighed amounts of thymol and polymer at different D:P weight ratios (2:1, 1:1, and 1:2 *w*/*w* %) were dissolved in 2 mL acetone and sonicated in an ultrasonic bath. The stabilizers (P-188/CTAB) were weighed and dissolved in 8 mL of distilled water. The organic:aqueous phase ratio was fixed at 1:4 in all formulations. The organic phase was then poured dropwise into the aqueous phase at a rate of 500 rpm employing magnetic stirring at 500 rpm. Thymol-loaded CPNPs directly appeared as a milky colloidal dispersion and were kept under stirring for 2 h to completely evaporate the acetone. Afterward, the as-prepared CPNPs were kept in closed glass vials until further use.

### 2.3. Characterization of Thymol-Loaded CPNPs

#### 2.3.1. Entrapment Efficiency Percentage (EE%)

Cooling centrifugation was used to separate the thymol from the thymol-loaded nanoparticles at 9000 rpm for 40 min at 4 °C (Union 32R; Hanil Co., Yongin, Gyeonggi-do, Republic of Korea). The centrifugation process was repeated twice to ensure that the drug was completely separated from the spaces between the nanoparticles. The amount of unentrapped thymol was determined in the supernatant using UV–Vis spectrophotometry (Shimadzu UV–Visible Spectrophotometer, 2401/PC; Tokyo, Japan) at 274 nm. Subtracting free drug quantity from the initial amount yielded the entrapped thymol amount.

The EE% was estimated employing the equation [17]:

$$EE\%=\frac{Total\;drug-Free\;drug}{Total\;drug\;}\times 100$$

#### 2.3.2. Particle Size (PS), Polydispersity Index (PDI), and Zeta Potential (ZP) Measurements

Dynamic light scattering (Nano ZS; Malvern Instruments, Malvern, UK) was employed to determine the PS, ZP, and PDI of the prepared CPNPs. The measurements were taken in a multimodal mode. All tests were carried out in triplicate.

#### 2.3.3. In Vitro Release Study

The In Vitro release study was accomplished by employing the dialysis bag-diffusion technique [30,31,32]. The dialysis method was previously reported to be is the most flexible and widely used approach for evaluating drug release from nano-sized dosage forms [33,34]. Briefly, a cellulose dialysis bag (Dialysis tubing cellulose membrane; Sigma Co., Ronkonkoma, NY, USA; molecular weight cutoff 12,000–14,000) was filled with an aliquot of the resuspended thymol-loaded CPNPs (equal to 2 mg thymol). The bags were tightly sealed at both ends and then dipped in 100 mL phosphate buffer saline (PBS), pH 7.4 [35]. The study was conducted at 100 rpm while maintaining the temperature at 32 ± 0.5 °C to mimic skin In Vivo conditions [36]. Withdrawals of sample took place at several time intervals and samples were further assayed spectrophotometrically at 274 nm. Fresh media was replaced at the same volume. The cumulative percentages of released thymol were determined in triplicate. The area under the release curve at time t (measured using the trapezoidal rule) was used to calculate the release efficiency percentages (R.E.%), which were then expressed as a percentage of the area of the rectangle corresponding to 100% release, for the same total time, using the following equation [37]:

$$R.E.\%=\frac{\int_{0}^{t}Y\times \;\mathrm{dt}}{\mathrm{Yt}\;\times 100}\times 100$$

where Y is the percentage of drug released at time t.

Various mathematical models were fitted to the drug release results, including zero-order, first-order, and the Higuchi and Peppas models, to study the drug-release mechanism. Linear regression analysis on the release data was performed and the regression coefficient (R2) was determined. The best fit model was determined with an R2 value near 1. According to Peppas theory, if the release exponent “n” ≤ 0.43, the drug is released via a Fickian diffusion process; if 0.43 < n < 0.85, the drug is released via non-Fickian diffusion; if n equals 0.85, it is released via case II transport; and if n > 0.85, it is released via super-case II transport [38].

#### 2.3.4. Morphology of Thymol-Loaded CPNPs

##### Transmission Electron Microscopy (TEM)

A drop of the prepared CPNPs was deposited on a carbon-coated copper grid. The sample was then left at room temperature for 5 min to dry. One % (*w*/*v*) phosphotungstic acid solution was used for staining, where a drop was put on the grid and left to dry. After that, the grid was placed into the TEM (JEOL Co., JEM-2100, Tokyo, Japan) and suitable micrographs were captured.

##### Scanning Electron Microscopy (SEM)

A Tescan SEM was used to conduct the scanning electron microscopy investigation (Tescan vega 3 SBU, Brno, Czech Republic). The examined formulations were carbon-taped to an aluminium microscope stub. A Q150t sputter coater was then used to coat the samples in gold (Au) for 180 s.

#### 2.3.5. Fourier Transforms Infrared (FT-IR)

Thymol, Eudragit RS-30D, P-188, CTAB, and thymol-loaded CPNPs were analyzed using a JASCO 6100 FT-IR spectrometer (JASCO, Tokyo, Japan). The examined samples were added to potassium bromide before being squeezed in a hydraulic press at 200 kg/cm^2^ for 3 min. Each sample was scanned counter to a blank.

#### 2.3.6. Powder X-ray Diffraction (PXRD)

The components of the developed formulations (Thymol, Eudragit RS-30D, P-188, CTAB, and thymol-loaded CPNPs) were analyzed by PXRD (Bruker AXS, D8 Advance, Bremen, Germany). The radiation source was the Kα line of copper, and the generator was run at a 40 kV tube voltage and 40 mA tube current.

### 2.4. In Vitro Mucoadhesion Study

The mucoadhesive characteristics of free thymol and optimized thymol-loaded CPNPs were studied by determining the ZP changes that happened upon contact of the examined formulations with mucin. The mucin dispersion (0.4 mg/mL) was mixed with the investigated samples in the same volume. The combinations were vortexed for a minute before the determination of their ZP values [39].

### 2.5. Incorporation in Hydrogels

The prepared thymol-loaded CPNPs were designed for the purpose of wound healing. Thus, they were incorporated into a hydrogel to be applied easily and to protect the developed thymol-loaded CPNPs from the environment. The selected thymol-loaded CPNPs were incorporated into methylcellulose (MC) polymer hydrogel bases in various concentrations (1, 2, and 3% *w/w*). The developed thymol-loaded CPNP pellets of each of the selected formulations (F5 and C2) were mixed with the MC hydrogels, with frequent stirring, until homogenous hydrogels of GF5 and GC2 developed, respectively. To get a translucent solution, the preparations were refrigerated for 48 h [40]. Free thymol hydrogel was prepared employing a solution of thymol dissolved in phosphate buffer (pH 7.4).

### 2.6. Characterization of the Developed Thymol-Loaded CPNPs-Based Hydrogel

#### 2.6.1. Physical Appearance

Visual observation was used to assess the prepared gels’ physical appearance and uniformity.

#### 2.6.2. Determination of pH

The pH values of the prepared gels were measured using a digital pH meter (Jenway, Bibby Scientific Limited, Staffordshire, UK), which was previously calibrated. Measurements were recorded in triplicate.

#### 2.6.3. Rheological Properties

The rheological properties of the developed hydrogels were evaluated using a parallel-plate rheometer (Anton Paar, Physica MCR 301, Ostfildern, Germany). A gradual increase in the shear rate was applied from 1 to 200 s^−1^ while recording the viscosity [41].

#### 2.6.4. In Vitro Release Study

The same procedure as that described in Section 2.3.3 was used. The release profile of thymol from the developed gels (GF5 and GC2) and the free thymol hydrogel was studied.

### 2.7. Cytotoxicity and Wound Healing Assay

Primary human adult dermal fibroblasts (HDFa, ATCC® PCS-201-012™), mouse macrophages (RAW 264.7) were used.

#### 2.7.1. Sulforhodamine B (SRB) Cell Cytotoxicity Assay

Cell viability was studied to investigate the IC_50_ of the developed formulations. The cytotoxicity of the developed formulations was studied against a normal human skin cell line (BJ-1), and cell viability was determined using the SRB assay [42]. Aliquots of 100 µL cell suspension (5 × 10^3^ cells), placed in a 96-well plate, were allowed to incubate in complete media for 24 h. Cells were treated with 100 µL media containing different concentrations of the solubilized drug formulations (free thymol hydrogel, GF5, and GC2) using Doxorubicin (DOX) as a positive control. Seventy-two hours post formulation exposure, cells were fixed by the replacement of media with 150 µL of 10% TCA and incubating for one hour at 4 °C. The TCA solution was then removed, followed by washing the cells with distilled water 5 times. A 70 µL aliquot of 0.4% *w/v* SRB solution was added, and the mixture was then left to incubate at room temperature for 10 min in the dark. The plates were air-dried overnight after being cleaned three times with 1% acetic acid. Then, 150 µL of TRIS (10 mM) was used to dissolve the protein-bound SRB stain [43]. Using a BMG LABTECH^®^ -FLUOstar Omega microplate reader (Otenberg, Germany), the absorbance was determined at 540 nm. GraphPad Instate software (version 6.01) was used to calculate the effective safe concentration (EC100) value (at 100% cell viability) of each investigated sample. The cytotoxicity rate (CT%) was calculated using the equation:

$$CT\left(\%\right)=\frac{\mathrm{AC}-\mathrm{At}}{\mathrm{Ac}\;\times 100}$$

where, Ac and At are the absorbance of the control sample and the test sample, respectively [44].

#### 2.7.2. In Vitro Cell Migration (Wound Healing) Assay

The effect of free thymol gel, GF5, and GC2 on the migration ability of epithelial cells was assessed using a protocol described by Mazutti [45]. Briefly, a monolayer of human skin fibroblast cell line was created by seeding at a density of 2 × 10^5^ per well onto a coated 12-well plate and overnight culturing in 5% FBS-DMEM at 37 °C and 5% CO_2_ [46]. The following day, horizontal scratches were made in the confluent monolayer. The plate was then washed thoroughly with PBS. In this experiment, human skin fibroblasts were subjected to sub IC_50_ (200 µg mL^−1^, 30 µg mL^−1^, and 50 µg mL^−1^) of the tested pharmaceutical formulations; free thymol hydrogel, GF5, and GC2, respectively. An inverted microscope was used to take photographs at predetermined intervals. Incubation of the plate took place at 37 °C and 5% CO_2_ in between the time points. The percentage of area reduction of wound closure, which rises as cells move over time, can be used to express the migration rate according to the following equation [47]:

$$\mathrm{Wound}\;\mathrm{closure}\;\left(\%\right)=\frac{A_{0}-\mathrm{At}}{A_{0}}\times 100\;$$

where A_0_ is the average size of the wound measured at the instant the scratch was made (time zero), and At is the average size of the wound measured h hours later.

### 2.8. In Vivo Skin Retention Studies

In Vivo skin-retention experiments of free thymol hydrogel, GF5, and GC2 were carried out on BALB/c mice (n = 3 per group). The backs of the mice (about 1 cm^2^) were shaved the day before the experiment. On the specific area of each rat, the formulations were applied (2 mg/cm^2^). After 4 h, the rats were euthanized with an overdose of anesthesia, followed by cervical dislocation, and their skin was dissected out. After washing the skin with Millipore water, the amount of thymol in a homogenized skin sample was measured by HPLC [48].

### 2.9. In Vivo Murine Model: Effect of Thymol-Loaded CPNPs-Based Hydrogel on the Staphylococcus Aureus Skin Infection

Infection of the skin was carried out in accordance with Hagras et al. [48]. Briefly, BALB/c mice were divided into five groups (n = 5/group), and their backs were shaven by a clipper. The mice were anesthetized using 2,2,2-tribromoethanol (25 μg/mL) and injected subcutaneously with 100 μL containing 4 × 10^9^ CFU of MRSA, suspended in sterile pyrogen-free saline. After this, food and water were continuously provided to the mice in their cages. On day 3, (72 h after infection), the site of infection was treated as follows: Group I was left untreated and acted as a control. Group II was treated with a drug-free vehicle (plain 3% MC hydrogel). Group III was treated with free thymol in 3% MC hydrogel. Groups IV and V were treated with the optimized formulations in 3% MC hydrogel of GF5 and GC2, respectively. The concentration of thymol in all the hydrogels was adjusted to 2%. The treatment was continued for 4 days, twice daily. On the seventh day, the mice were euthanized with an overdose of anesthesia, followed by cervical dislocation. Each mouse’s spleen and a skin patch equivalent to 1.5 cm^2^ around the lesion site were aseptically removed. A sterile surgical blade was used to shred the skin patch, which was subsequently homogenized in 1 mL of pyrogen-free saline. One mL of pyrogen-free saline was used to homogenize the spleen. Following serial dilution, the homogenates were plated on mannitol salt agar (MSA) plates for colony counts.

### 2.10. Statistical Analysis

Statistical analysis was performed using GraphPad Prism (version 6.0) (GraphPad Software, Inc., La Jolla, CA, USA), employing the one-way ANOVA, followed by Dunnett’s multiple comparisons test. A *p*-value less than or equal to 0.05 was considered statistically significant.

## 3. Results and Discussion

### 3.1. Preparation of Thymol-Loaded Polymeric Nanoparticles

Thymol-loaded CPNPs were fabricated using the nanoprecipitation method employing two cationic polymers (Eudragit RL30D and Eudragit RS30D) and two stabilizers (P-188 and CTAB) at different D:P weight ratios (2:1, 1:1, and 1:2 *w*/*v*%). The thymol-loaded CPNPs appeared as a milky colloidal dispersion and were further characterized via several characterization methods. The composition and weight ratios of the different thymol-loaded nanoparticles are given in Table 1.

### 3.2. Characterization of Thymol-Loaded Nanoparticles

#### 3.2.1. Drug Entrapment Assessment

Entrapment efficiency (EE%) is a vital parameter that determines the amount of drug entrapped in the developed nanosystem. The EE% of thymol in the prepared CPNPs was relatively high (56.58–68.97%) (Table 1). It was found that a D:P ratio of (1:1) exhibited the highest EE% among the F1 to F6 formulations; thus, this ratio was selected for investigating the effect of the inclusion of CTAB at a concentration of 0.25 (*w/v*%). The results also reveal that the EE% of the Eudragit RL30D nanoparticles was higher than that of the Eudragit RS30D nanoparticles. In general, Eudragit RL materials contain higher percentages of quaternary ammonium groups than Eudragit RS materials. These groups make drug diffusion easier during the preparation of nanoparticles [49]. Similar results were reported previously [50,51]. A number of parameters can also affect drug EE% in nanoparticles, including low drug solubility in the aqueous phase, quick polymer precipitation in the aqueous phase, the low viscosity of the internal phase, and drug solubility in the polymer [52,53].

The results show that, for both types of polymers employed, increasing the concentration of polymer (changing the D:P ratio from 2:1 to 1:1) revealed a significant increase (*p* ≤ 0.05) in EE%. This finding could be explained by the organic phase’s increased viscosity, which led to an increased drug-molecule diffusional resistance while migrating from the organic to the aqueous phases, resulting in an increase in EE% [54]. Further increases in polymer concentration (changing the D:P ratio from 1:1 to 1:2) revealed an insignificant decrease in EE%. The same effect was observed after the inclusion of the cationic stabilizer CTAB (formulations C1 and C2). The higher polymer concentration created a more compact polymer coat, preventing adequate drug entrapment [55].

#### 3.2.2. Particle Size, ZP, and PDI of the Developed Thymol-Loaded CPNPs

The characterization results (Table 1) revealed that the PS of all the prepared formulations were on the nanoscale, ranging from 36. 30 ± 2.02 to 99.41 ± 3.51 nm. It could be seen that increasing the polymer concentration led to an increase in PS for both polymers employed; where PS values of F3 > F2 > F1 and F6 > F5 > F4 for the Eudragit RL30D- and Eudragit RS30D-based CPNPs, respectively. The increasing viscosity of the polymer organic phase solution prevents the nanoparticles from being dispersed in the aqueous phase, resulting in the formation of particles with larger sizes. These findings match previous reports [17,56]. The inclusion of CTAB in formulations C1 and C2 led to a significant increase (*p* ≤ 0.05) in PS, compared to F2 and F5, respectively. It was reported that increasing stabilizer concentration results in higher interactions of stabilizer molecules, and, thus, more adsorption onto the surface of the particles and the development of multiple layers having larger sizes [57]. Similar findings were previously reported [56,58]. PDI is a vital parameter that determines the distribution pattern of the formulation. PDI value of ≤0.5 indicates homogenous and narrowly distributed dispersion [14,59]. The PDI of the developed thymol-loaded CPNPs were in the range of 0.216 to 0.489, indicating homogeneity as well as a narrow distribution of the dispersed particles [60].

Zeta potential determines the stability of a material. The results reveal that all the developed formulations exhibited positively charged ZP values (Table 1). These positively charged values might be related to the quaternary ammonium groups present in the employed cationic polymers (Eudragit RL30D and Eudragit RS30D). It was reported that the aggregation of particles is negligible for particles with absolute ZP values of more than 20 [61]. ZP values of the investigated formulations ranged from 20.10 ± 2.21 to 25.5 ± 1.35 mV, revealing their physical stability. It was noticed that increasing the polymer concentration led to a slight increase in ZP values. Also, the inclusion of the cationic stabilizer CTAB (C1 and C2) resulted in a significant increase in the positively charged ZP values, compared with F2 and F5, respectively. A positive charge on nanoparticles or macromolecules seems to enhance gene transfer, imaging, and drug delivery efficacy, in general. The formulations with a D:P ratio of (1:1) (F2 and F5) were selected for further investigations because they had the highest EE%, in addition to their appropriate PS and ZP values. C1 and C2 were also further studied for investigation of the CTAB-inclusion effect.

#### 3.2.3. In Vitro Release Profiles of the Developed Thymol-Loaded CPNPs

**Figure 1:**
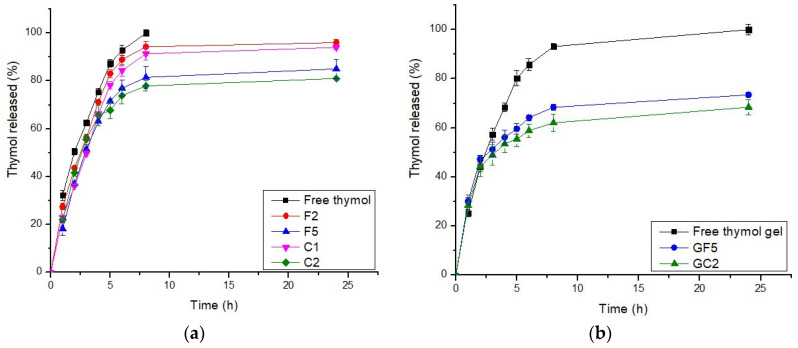
In Vitro release profiles of thymol from (**a**) thymol-loaded CPNPs and (**b**): thymol-loaded CPNP hydrogels (GF5 and GC2). (n = 3).

In general, an effective drug carrier should have a better binding efficiency and a longer release time. The drug delivery system should include a sufficient amount of drug that could be released at the site of action for a longer period of time, reducing the dose and frequency of administration [62]. The drug-release profiles from the selected thymol-loaded CPNPs are presented in Figure 1a. These profiles reveal that thymol release from the developed CPNPs was more sustained up to 24 h, compared to free thymol. There was no burst release, showing that thymol was incorporated in the polymer matrix rather than just adsorbed on the surface. This is in accordance with previous studies [17,63]. The calculated R.E. % (Table 1), revealed that thymol release from free thymol and developed CPNPs was in the following pattern: free thymol (89.59) > F2 (85.93) > C1 (81.34) > F5 (73.71) > C2 (72.59). Thus, it was concluded that drug release from the cationic polymer Eudragit RS30D exhibited a higher sustained release than that from Eudragit RL30D. This was attributed to the difference in the degree of hydrophobicity between the two polymers, as the hydrophobicity of Eudragit^®^ RS30D is higher than that of Eudragit^®^ RL30D [64]. Thus, the hydrophobic thymol is more attached to Eudragit RS30D leading to enhanced sustained drug release. It could be also seen that the inclusion of CTAB resulted in a higher sustained release effect for both polymers employed. This might be attributed to the existence of electrostatic interaction between the hydroxyl group of thymol and the ammonium moiety in CTAB, as will be discussed in the FT-IR studies. The hydrogen bonds formed between CTAB and thymol, as shown in the FT-IR spectra, has led to a higher sustained release profiles of C1 and C2, compared to F2 and F5, respectively. In conclusion, F5 and C2 exhibited higher sustained release profiles, thus, they were selected for further investigations.

The release kinetic order analysis of thymol from the developed CPNP formulations was studied. The release data did not show appropriate fitting to zero order, first order, or the Higuchi model. Fitting the release pattern to the Peppas model showed better clarification of the drug release order. Thus, the drug-release profile data were fitted to the Peppas model, and the release exponent value “n” was calculated for each formulation. The n values ranged from 0.43 to 0.85, suggesting an anomalous drug-release pattern that is governed by many mechanisms including drug diffusion, relaxation, and erosion.

#### 3.2.4. Morphological Properties of Thymol-Loaded CPNPs

**Figure 2:**
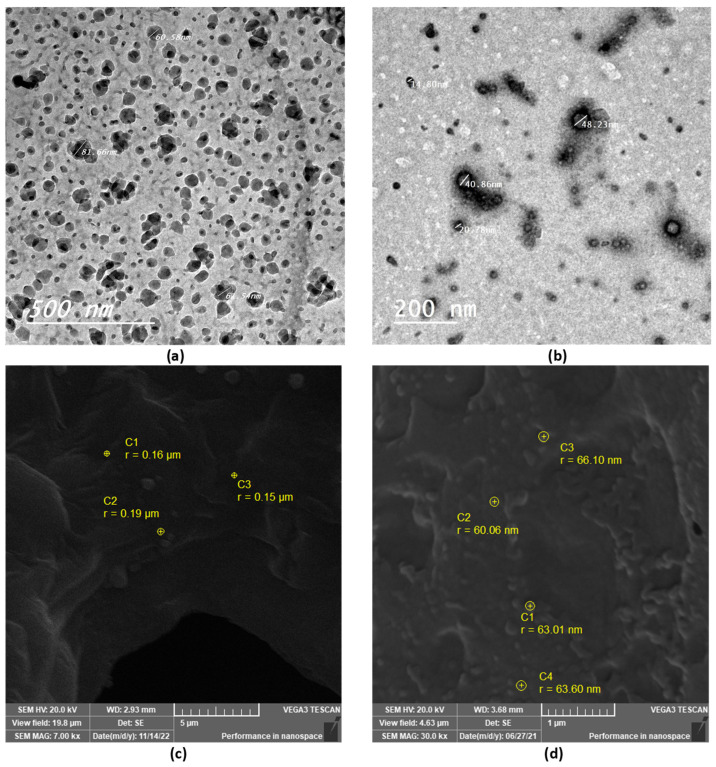
TEM and SEM micrographs of selected thymol-loaded CPNPs; F5 (**a**,**c**) and C2 (**b**,**d**).

TEM and SEM were used to examine the morphological properties of the selected thymol-loaded CPNPs. The spherical shape of the thymol-loaded CPNPs was seen in the TEM micrographs (Figure 2a,b). The particles were nano-sized and scattered evenly, with no agglomeration. The spherical shape of the CPNPs was further confirmed by the SEM micrographs (Figure 2c,d), where the developed CPNPs exhibited a spherical shape with a smooth surface and uniform texture.

#### 3.2.5. FT-IR

**Figure 3:**
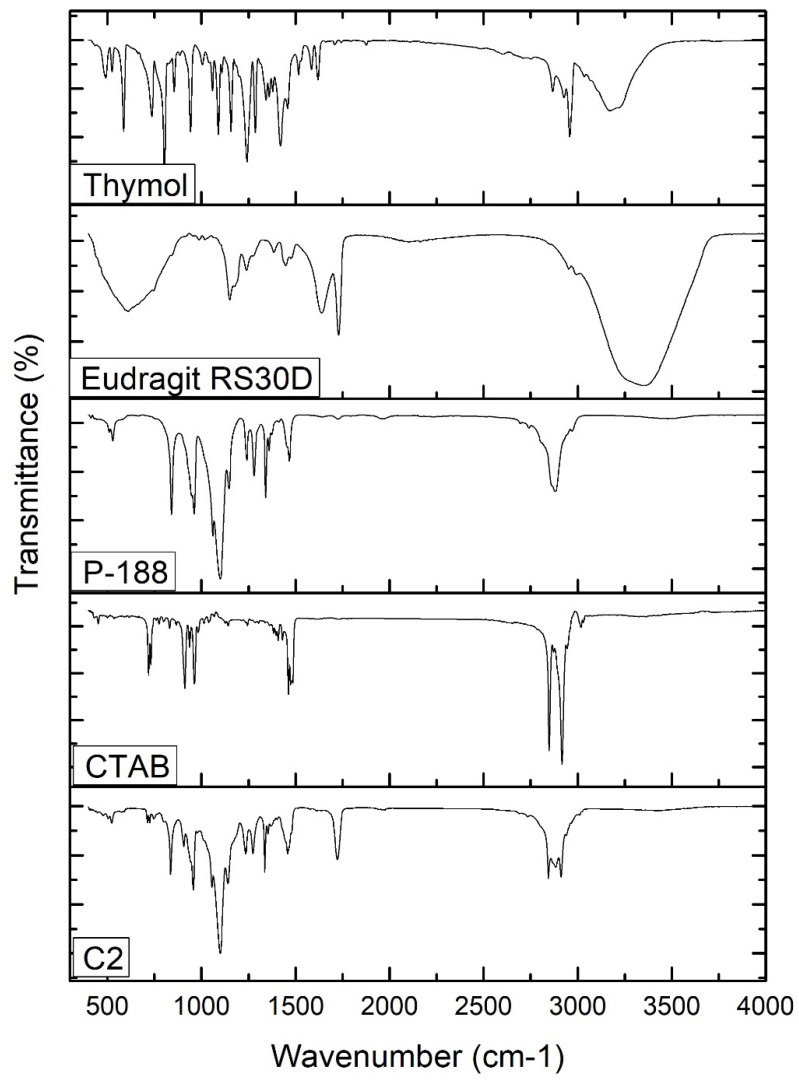
FT-IR spectra of thymol, Eudragit RS30D, P-188, CTAB, and thymol-loaded CPNPs C2.

The FTIR spectra of free thymol, P-188, Eudragit RS30D, CTAB, and thymol-loaded CPNP formulation C2 were analyzed (Figure 3). The FT-IR spectrum of thymol revealed characteristic peaks at 3035 and 3170 cm^−1^, related to =C-H stretching of aromatics. The peaks observed at 1285 cm^−1^ and 2926 cm^−1^ correspond to vibrations of C-O and -CH_3_, respectively. The characteristic peaks observed at 1620 cm^−1^, 1585 cm^−1^, and 1458 cm^−1^ correspond to C=C stretching in aromatics. C-H bending was recorded at 1089 cm^−1^ and 1058 cm^−1^. These stretching peaks of thymol match those of previous studies [54]. The P-188 spectrum revealed characteristic peaks at 3480 cm^−1^, 2878 cm^−1^, and 1095 cm^−1^, corresponding to the O-H group, C-H group, and C-O group, respectively. The Eudragit RS 30D spectrum showed a peak at 2953 cm^−1^, attributed to C-H stretching, bands at 1150 cm^−1^ and 1239 cm^−1^, corresponding to the ester groups, and a peak at 1728 cm^−1^ corresponding to the C=O stretching ester vibration. The FT-IR spectrum of CTAB revealed a broad band at 3325 cm^−1^ that was attributed to the ammonium group stretching vibrations. Peaks at 2916 cm^−1^ and 2849 cm^−1^ are attributed to the symmetric and asymmetric C-H band vibrations of the -CH_2_ group, respectively. The band at 1473.89 cm^−1^ corresponds to the stretching vibration of N^+^-CH_3_. Out-of-plane -CH vibration of CH was observed as a band at 961.33 cm^−1^. Similar absorption bands of the FTIR spectrum of CTAB were previously reported [55,56,57].

The thymol-loaded CPNP formulation (C2) spectrum exhibits slightly shifted characteristic peaks of P-188, namely, 3429.95 cm^−1^, 2889.02 cm^−1^, and 1060.72 cm^−1^, as well as peaks of Eudragit RS30D; namely, 1146.19 cm^−1^, 1240.84, and 1728.53 cm^−1^. The spectrum also revealed a slight shifting of the distinctive peaks of CTAB, namely, 2917.18 cm^−1^, 2849.81 cm^−1^, 1463 cm^−1^, and 962.18 cm^−1^. The broad band at 3429.95 cm^−1^ was attributed to the electrostatic interaction between the thymol hydroxyl group and the CTAB ammonium moiety in CTAB [65]. The distinctive peaks that were observed for the free thymol were not present in the thymol-loaded CPNP formulation spectrum, revealing complete encapsulation of thymol in the developed CPNPs. Hence, FT-IR analysis confirmed the development of thymol-loaded CPNPs (C2) comprising P-188, Eudragit RS30D, and CTAB.

#### 3.2.6. PXRD

**Figure 4:**
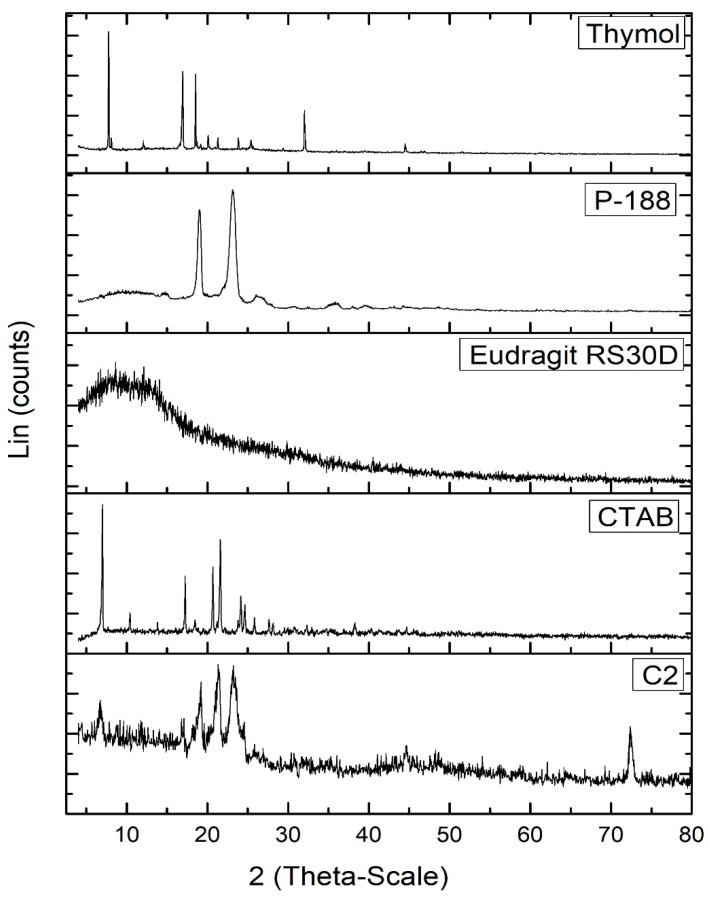
PXRD diffractograms of thymol, Eudragit RS30D, P-188, CTAB, and thymol-loaded CPNPs C2.

The PXRD diffractogram of free thymol (Figure 4) exhibited many crystalline peaks; at 7.75°, 12.07°, 16.65°, 19.14°, 20.08°, 20.81°, 21.28°, 23.85°, 32.01°, and 44.49° 2θ. These peaks revealed the crystalline nature of thymol [66]. The P-188 XRD diffractogram showed two peaks at 19.14° and 23.25° 2θ. The Eudragit RS30D diffractogram revealed broad peaks at 8.13° and 12.49 2θ, indicating its amorphous state. The XRD diffractogram of CTAB revealed the periodical main peaks at 2θ = 7.005°, 10.42°, 13.81°, 17.09°, 20.69°, 24.14°, 27.60°, and 31.10° of CTAB, indicating its crystalline nature. Most characteristic peaks of P-188, Eudragit RS30D, and CTAB disappeared from the XRD diffractogram of the thymol-loaded CPNPs (C2). Furthermore, the distinctive peaks for thymol disappeared in the thymol-loaded CPNPs, revealing the entrapment of thymol inside the lipid core in an amorphous form [67].

#### 3.2.7. In Vitro Mucoadhesion Study

**Figure 5:**
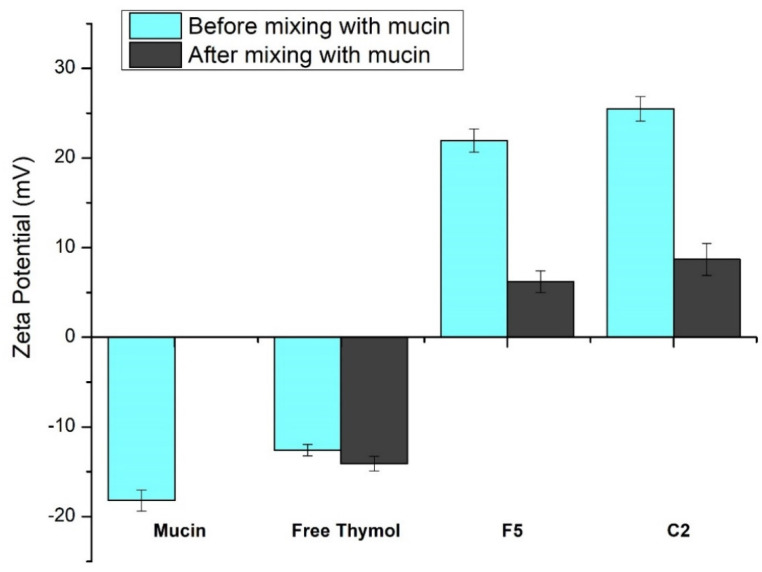
Zeta potential values obtained for mucin and free thymol as well as thymol-loaded CPNPs (F5 and C2) before and after mixing with mucin. Data are displayed as means ± S.D. (n = 3).

Figure 5 demonstrates the effect of mucin on the ZP value of free thymol and the developed thymol-loaded CPNPs. The glycoprotein mucin is found in the mucus membrane. Due to the negatively charged sialic acid, mucin serves as an anionic electrolyte [68]. Mucin dispersion and free thymol revealed ZP of negative values, −18.2 ± 1.18 mV and −12.6 ± 0.63 mV, respectively. After mixing thymol suspension and mucin, this led to an insignificant change in thymol ZP value (−14.1 ± 0.82 mV). Zeta potential values of the prepared thymol-loaded CPNPs (F5 and C2) showed a significant decrease (*p* ≤ 0.05) from 21.95 ± 1.29 and 25.5 ± 1.35 to 6.2 ± 1.21 and 8.7 ± 1.78 mV, respectively, following mucin mixing. This could be explained by the presence of ionic interaction between the positively charged Eudragit RS30D (in F5 and C2) as well as the cationic CTAB (in C2) with mucin’s anionic sialic acid residues. Furthermore, poloxamers have mucoadhesive characteristics and stabilizing impact. The mucoadhesive effect of poloxamers is based on the effects of the double lipophilic and lipophobic components of the poloxamers, which are analogous to mucosal action [69]. These results are consistent with findings from earlier investigations [70,71].

### 3.3. Preparation of Topical Thymol-Loaded CPNPs Hydrogel

Thymol-loaded CPNPs hydrogels were successfully developed employing MC polymer. Dispersions of MC gel bases in various concentrations (1, 2, and 3% *w*/*w*) were investigated. The best degree of gelation was established upon using MC at 3% *w*/*w*. Thus, this content was selected for adequate preparation of CPNP hydrogels.

### 3.4. Characterization of the Developed Topical Formulation

#### 3.4.1. Physical Properties

The physical properties of the developed thymol-loaded CPNP hydrogel were appropriate for pharmaceutical use. For all the prepared gels, the color was transparent-to-mild-white with no phase separation, with a translucent appearance and smooth consistency after application. All the produced gels were homogenous and washable, with no aggregation or harsh particles. These properties were in accordance with the model requirements for a topical gel [40].

#### 3.4.2. Measurement of pH

The pH of the developed gel formulations was 5.41 ± 0.12 and 5.82 ± 0.09 for GF5 and GC2, respectively. Both values represent an appropriate pH because they are not very different from the pH of the human skin [72]. Furthermore, this pH does not irritate the skin but does stabilize the gel’s rheological behavior [73].

#### 3.4.3. Rheological Properties

**Figure 6:**
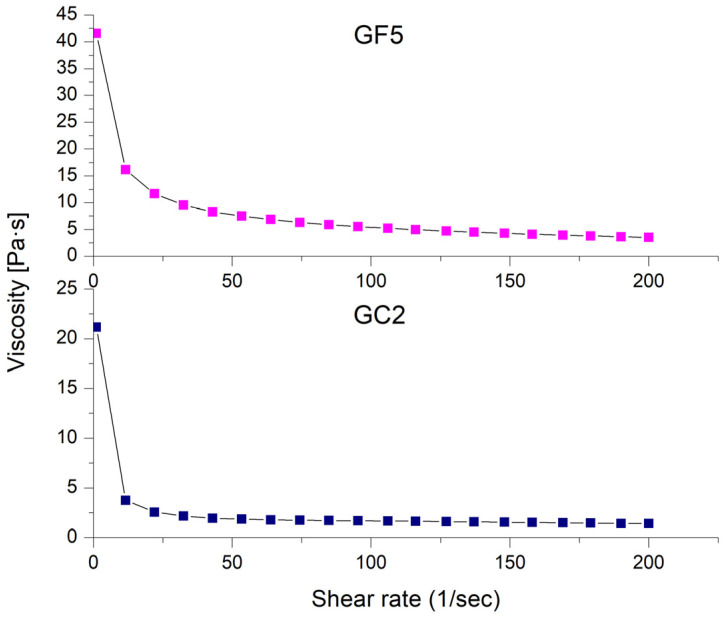
Shear-rate dependence of viscosity for the selected thymol-loaded CPNP hydrogels (GF5 and GC2).

The rheological properties of the prepared thymol-loaded CPNP hydrogels (GF5 and GC2) are shown in Figure 6 as the shear rate plotted against viscosity. The rheological features of both formulations investigated (GF5 and GC2) revealed a pseudoplastic flow (shear-thinning behavior). At low shear rates, the viscosity increased, while it decreased at high shear rates. Shear-thinning systems have the advantage of showing increased viscosity upon application, which is favored economically [74].

#### 3.4.4. In Vitro Release Profiles of the Developed Thymol-Loaded CPNP Hydrogels

The drug release profiles from free thymol in hydrogel as well as the selected thymol-loaded CPNP hydrogels (GF5 and GC2) are presented in Figure 1b. It was concluded that the drug release from the GF5 and GC2 occurred in two phases, a phase characterized by a relatively moderate drug release rate, followed by a steady phase with a more sustained and controlled release, which was maintained for 24 h. The cumulative percentages of thymol released from the developed CPNPs after 24 h were 73.45% and 68.40% for GF5 and GC2, respectively, revealing a sustained release effect compared to free thymol hydrogel (100%). The R.E. values for free thymol, thymol hydrogel, GF5, and GC2 were 89.59, 86.00, 64.13, and 59.35 %, respectively. Statistical analysis revealed significant differences (*p* ≤ 0.05) between the R.E.% of free thymol and thymol hydrogel as well as between thymol hydrogel and both developed formulae (GF5 and GC2). The results also reveal that thymol release from the hydrogels was more sustained compared to that from their corresponding CPNPs (Figure 1a). This was attributed to the higher viscosity of the gel matrix.

### 3.5. Cytotoxicity on Normal Human Skin Cell Line

**Figure 7:**
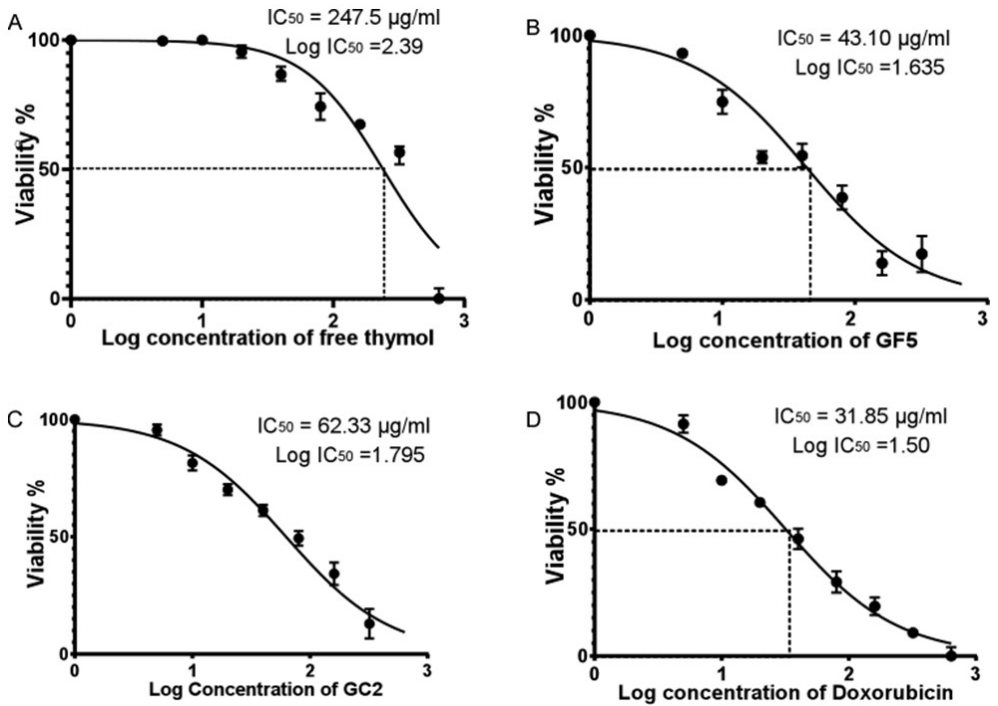
Half maximal inhibitory concentration (IC_50_) of free thymol hydrogel, GF5, GC2, and Doxorubicin against normal human skin cell line (BJ-1) using the SRB colorimetric assay. Figures (A–D) represent the percentage of cell viability of BJ-1 cells against different concentrations of free thymol, GF5, GC2 and Doxorubicin, respectively. Error bars represent the standard error. A *p* value < 0.05 was considered significant.

In this study, the Sulforhodamine B (SRB) assay was performed employing a normal human skin cell line (BJ-1) to evaluate the cytotoxicity of free thymol hydrogel, GF5, and GC2. The SRB assay was applied to detect the percentage of cell viability of BJ-1 cells against different concentrations of free thymol hydrogel, GF5, and GC2 (5, 10, 20, 40, 80, 160, 320, and 640 µg/mL). At 48 h post drug treatment, a consistent decrease in cell viability of BJ-1 cells was observed with the increase in the concentration of the tested formulations. Results showed that the three tested formulations significantly inhibited proliferation in the BJ-1 cell line by exhibiting cytotoxic effects (Figure 7). The IC_50_ values for free thymol hydrogel, GF5, and GC2 were calculated to be 247.5 µg.mL^−1^, 43.1 µg.mL^−1^, and 62.33 µg.mL^−1^, respectively. In addition, cells treated with doxorubicin, serving as a positive control in this study, showed significant inhibition in the proliferation of BJ-1 cells (IC_50_ = 31.85 µg.mL^−1^) in a dose-dependent manner.

The lower IC_50_ of the developed thymol CPNP hydrogels (GF5 and GC2) could be justified by their small particle size and wide surface area, which facilitate the CPNPs’ easy entry into the nucleus and subsequent DNA damage and apoptosis [75]. Cationic nanoparticles impair plasma membrane integrity more markedly, produce more mitochondrial and lysosomal injury, and cause a higher number of autophagosomes [76]. Under osmotic stress, the cationic agents are assumed to interact with the components of phospholipid in the cytoplasmic membrane, inducing membrane modification and protoplast lysis [77]. The developed CPNPs have amino moieties in their structures that, when ionized in a physiological context, would strongly interact with a negatively charged cell membrane. This adhesion may lead to cell death and lysis [78]. The presence of quaternary ammonium groups in Eudragit RS30D, employed in the preparation of GF5 and GC2, is considered to be the main moiety producing cytotoxicity. The quaternary ammonium compounds led to the occurrence of positive surface charge on the developed formulations. This charge exhibits a significant impact on their cytotoxic effect because of the electrostatic ionic interaction between the cells’ negatively charged groups and the positively charged ammonium moieties of Eudragit [79].

### 3.6. Cell Migration Assay (Wound Scratch Assay)

**Figure 8:**
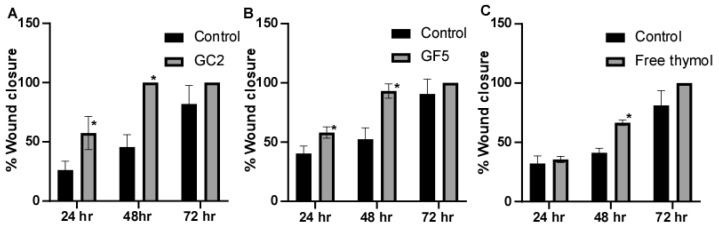
Wound-closure effect of (**A**) GC2, (**B**) GF5, and (**C**) free thymol hydrogel on the wound-healing process compared with the untreated group. Mean values of three independently produced experiments ± S.E. * indicates significant difference at *p* < 0.05, compared to control group.

The ability of the pharmaceutical formulations (free thymol hydrogel, GF5, and GC2) to promote fibroblast migration was assessed using the classic In Vitro cell migration assay. In this experiment, human skin fibroblasts were subjected to sub IC_50_ of the tested pharmaceutical formulations; free thymol gel, GF5, and GC2, respectively. Results showed that the three tested pharmaceutical formulations increased cell regeneration and possess wound-healing activity compared to the control (Figure 8). For the formula GC2, fibroblasts migration was significantly (*p* ≤ 0.05) enhanced by 57.4% 24 h, and 100% 48 h, post exposure to GC2, compared to the negative control which showed an enhancement of only 26.14% after 24 h and 45.57% after 48 h. In addition, the GF5 formula showed a significant (*p* ≤ 0.05) acceleration in the wound closure at 24 h (58.09%) and 48 h (93.15%) compared to the negative control (40.40% after 24 h and 52.50% after 48 h). In addition, they demonstrated a better contraction percentage compared to that of the negative control group after 48 h. Regarding the free thymol, no significant difference was observed after 24 h compared with the negative control. Eventually, after 72 h, the three tested formulations did not show a significant difference in impact on the healing process compared to the negative control.

Figure 7A–D represent the percentage of cell viability of BJ-1 cells against different concentrations of free thymol hydrogel, GF5, GC2, and Doxorubicin, respectively. IC50 for each drug formula was calculated. Statistical analysis was performed using GraphPad Prism (version 6.0) (GraphPad Software, Inc., USA), applying the one-way ANOVA. Error bars represent the standard error. A *p*-value < 0.05 was considered significant.

### 3.7. In Vivo Skin-Retention Studies

The In Vivo skin retention of thymol was assessed after the application of 2 mg/cm^2^ of free thymol hydrogel, as well as optimized thymol-loaded CPNP hydrogels (GF5 and GC2), on hairless mice. The results revealed that the skin retained an amount of thymol (62.61 ug/cm^2^), 4 h post application of free thymol hydrogel. It could be concluded that both examined formulations of thymol-loaded CPNP hydrogels (GF5 and GC2) significantly (*p* ≤ 0.05) improved skin retention of thymol at 4 h post-application (Table 2). Incorporating thymol in the developed CPNP hydrogels (GF5 and GC2), resulted in 3.3- and 3.65-fold higher concentrations of thymol, respectively, compared to free thymol hydrogel. These findings agree with the In Vivo results where the GF5 and GC2 showed a higher wound-healing effect compared to the free drug. Polymeric nanoparticles can work as a local drug depot once they have accumulated at the target site. Thus, they provide a source for the continuous supply of the encapsulated therapeutic compound(s) at the site of action [80].

### 3.8. In Vivo Murine Model: Effect of Thymol-Loaded CPNPs-Based Hydrogel on the Staphylococcus Aureus Skin Infection 

**Figure 9:**
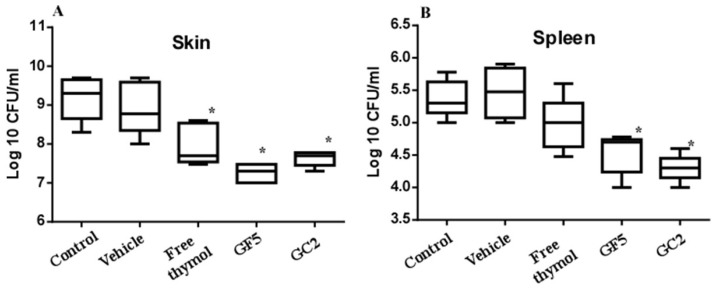
Effect of free thymol hydrogel, GF5, and GC2 on *S. aureus* murine skin infection. (**A**) Box plot of the bacterial burden recovered from skin lesions of mice. (**B**) Box plot of the bacterial burden recovered from the spleen. Statistical analysis was performed using GraphPad Prism (version 6.0) (GraphPad Software, Inc., USA), applying the one-way ANOVA, followed by Dunnett’s multiple comparisons test. Error bars represent the standard error. The * indicates significant difference at *p* value ≤ 0.05, compared to control group.

The murine skin model was adopted since skin abscesses represent a hallmark of staphylococcal infections [11]. The three pharmaceutical formulations (free thymol hydrogel, GF5, and GC2) were tested for their ability to combat MRSA infection in the murine skin model. Skin lesions were accomplished in all mice within 72 h post subcutaneous injection with MRSA. Mice were either left untreated (negative control) or treated with the free thymol hydrogel, GF5 or GC2, formulations, at the same thymol concentration (2%). The bacterial counts recovered from the skin lesions of the mice and the bacterial dissemination (reflected by detecting the bacterial count recovered from the spleen) were assessed. The bacterial counts recovered from the skin lesions of the mice groups treated with the free thymol, GF5, or GC2, were significantly (*p* ≤ 0.05) reduced compared with the counts recovered from the lesions of the negative control group (Figure 9). The bacterial counts, recovered from the spleens of either the GF5- or GC2-treated groups, were significantly (*p* ≤ 0.05) lower than the counts recovered from the spleen of the negative control group; however, no significant difference was found between the bacterial counts recovered from the spleen of the group treated with free thymol preparation (*p* ≤ 0.05).

It is worth noting that, in recent years, encapsulation of natural drugs in nanoparticle systems has been well documented for the innovation of new therapeutic agents with enhanced therapeutic effectiveness [81,82]. In recent years, encapsulation of thymol in nanoparticle systems has emerged as an innovative and promising alternative that enhances the well-documented therapeutic effectiveness of thymol [36,83,84,85]. In a just-emerging study, significant concentration-dependent reductions in MRSA viability and adhesion were observed in response to thymol treatment. In addition, thymol exposure also inhibited MRSA biofilm formation and enhanced the eradication of MRSA-preformed mature biofilms [86].

The current study calls attention to the potential of thymol nano-formulated topically applied preparations to combat MRSA skin infection. Detailed studies on the potential of the newly developed GF5 or GC2 formulations in the management of resistant bacteria causing skin infections, such as *Pseudomonas aeruginosa* and *Acinetobacter baumanii*, is highly recommended.

## 4. Conclusions

Thymol-loaded CPNPs were fabricated successfully using a nanoprecipitation method employing different weight ratios of thymol:polymer and were further characterized. The developed formulations exhibited average-to-high EE%, suitable PS, and positively charged ZP ≥ 20. Thymol was sustained-released from the developed CPNPs for up to 24 h, according to In Vitro release experiments. Morphological analysis, performed by TEM and SEM, showed the smooth surfaces and spherical shape of the prepared CPNPs. The FT-IR spectral study supported the contention that the thymol had been successfully loaded into the CPNPs. PXRD showed that thymol was found to be confined in the lipid core in an amorphous state. The developed thymol CPNPs’ mucoadhesive activity was confirmed by In Vitro mucoadhesion experiments. Selected thymol CPNPs (F5 and C2) were mixed with 3% methylcellulose to develop hydrogels for topical application. The developed hydrogels (GF5 and GC2) were characterized for their physical appearance, pH, rheological characteristics, and In Vitro drug release. Applying GF5 and GC2, resulted in 3.3- and 3.65-fold higher skin retention, respectively, compared with free thymol hydrogel. In Vitro and In Vivo models revealed the remarkable potential of GF5 and GC2 in managing wound healing and combating MRSA infections. This study presents thymol nano-formulated hydrogels as a new therapeutic opportunity with effective skin retention and wound-healing efficacy for topical use. The developed thymol-loaded cationic polymeric nanoparticle-based hydrogels have a significant added value in the management of skin abscesses in the era of MRSA.

## Figures and Tables

**Table 1:** Composition and characterization parameters of the prepared thymol-loaded polymeric nanoparticles.

Formula Code	Drug: Polymer (w/w%)	Polymer	EE% ± S.D. (%)	PS ± S.D. (nm)	PDI	ZP ± S.D. (mV)	R.E. ± S.D. (%)
F1	2:1	Eudragit RL30D	60.32 ± 2.84	36.30 ± 2.02	0.477	20.10 ±2.21	-
F2	1:1		68.97 ± 2.70	42.91 ± 1.31	0.489	20.92 ± 2.44	85.93 ± 1.79
F3	1:2		67.67 ± 1.56	88.97 ± 1.82	0.421	22.01 ± 2.05	-
F4	2:1	Eudragit RS30D	56.58 ± 2.02	44.45 ± 2.65	0.408	21.82 ± 2.71	-
F5	1:1		64.82 ± 2.08	49.81 ± 2.12	0.483	21.95 ± 1.29	73.71 ± 1.86
F6	1:2		62.19± 1.45	53.34 ± 3.01	0.451	22.31 ± 2.25	-
C1 *	1:1	Eudragit RL30D	64.32 ± 2.31	98.1 ± 3.25	0.216	23.37 ± 3.31	81.34 ± 1.83
C2 *	1:1	Eudragit RS30D	63.17 ± 1.22	99.41 ± 3.51	0.372	25.5 ± 1.35	72.59 ± 2.87

P-188 was fixed as 0.5 (*w*/*v*%) in all formulations; * CTAB was included in formulations C1 and C2 at a concentration of 0.25 (*w*/*v*%); Data are expressed as mean ± SD (n = 3).

**Table 2:** Thymol amount retained in ski 4 h post-application of free thymol hydrogel and thymol loaded PNPs gels (GF5 and GC2).

Groups	Thymol Retained (ug/cm^2^)(Mean ± S.D.)
Free thymol hydrogel	62.61 ± 8.15 ^a^
GF5	206.99 ± 9.55 ^b^
GC2	228.97 ± 0.76 ^b^

The same letter means non-significant difference, while a different letter means significant difference at *p* ≤ 0.05.

## Data Availability

Not applicable.
